# Cannabis at the End of Life: A Road More Traveled

**DOI:** 10.1093/geroni/igab046.1968

**Published:** 2021-12-17

**Authors:** J Alton Croker

**Affiliations:** CTCRE, CVRI, UCSF, Chicago, Illinois, United States

## Abstract

This study examines medical cannabis as a complement or alternative to palliative care (PC). Using cross-sectional survey data from 708 terminal patients in the Illinois Medical Cannabis Program, we compare those in PC (n = 115) to those who are not (n = 593). Increased odds of PC utilization were observed for prior military service, cancer diagnosis, low psychological wellbeing, and medical complexity. PC was positively associated with improvement scores for pain, and ability to manage health status. Higher pain levels were also observed for PC patients who indicated concurrent use of cannabis and opioids, compared to those not using opioids. While most terminal patients use cannabis as an alternative to PC, medical cannabis does operate as a therapeutic complement for individuals in PC to help manage pain and overall health status, and is used at higher levels of pain when patients are also using opioids.

